# Personality Traits, Stress, Anxiety, Depression Levels, Fear of Childbirth, and Affecting Factors in Turkish University Students

**DOI:** 10.1155/da/5130737

**Published:** 2025-06-03

**Authors:** Nülüfer Erbil, Hilal Gül Boyraz Yanık, Gizem Yıldız

**Affiliations:** Department of Obstetrics and Gynecologic Nursing, Faculty of Health Sciences, Ordu University, Ordu, Türkiye

**Keywords:** anxiety, depression, fear of childbirth, personality type, stress, university student

## Abstract

**Objectives:** This study was conducted to investigate the personality traits, stress, anxiety, depression levels, fear of childbirth, and affecting factors in Turkish female university students.

**Materials and Methods:** The study was planned as a descriptive and correlational type. The sample consisted of 500 female students who met the research criteria at the Faculty of Health Sciences and Faculty of Education of a university. The data were collected face-to-face using a personal information form, including socio-demographic and pregnancy-birth-related thoughts, the Childbirth Fear–Prior to Pregnancy (CFPP) scale, the Type D Personality Scale (negative affectivity and social inhibition subscales), and the Depression Anxiety Stress Scale-21 (DASS-21). Ethics committee approval and institutional permissions were obtained from the students to conduct the study. In the analysis of the data, the Kolmogorov–Smirnov test, descriptive methods Mann–Whitney *U*, and the Kruskal–Wallis test were used.

**Results:** 52.8% of the students were from the Faculty of Health Sciences, 48.4% were nursing students, and 30.6% were in their first class of students. The mean CFPP score was 40.14 ± 11.35. The mean score was 12.01 ± 6.79 for negative affectivity, 9.58 ± 6.09 for social inhibition, 6.75 ± 4.05 for stress, 5.28 ± 4.09 for anxiety, and 5.58 ± 4.50 for depression. There was a low level of positive correlation between CFPP and social inhibition subscales (*r* = 0.113), negative affectivity (*r* = 0.282), stress (*r* = 0.241), anxiety (*r* = 0.231), and depression (*r* = 0.221 (*p* = 0.01). The predictor of students' fear of childbirth prior to pregnancy was negative affectivity.

**Conclusions:** Turkish university students' fear of childbirth prior to pregnancy was associated with personality traits, depression, anxiety, and stress. It is recommended to identify the depression, anxiety, stress levels, personality traits, and fear of childbirth of female university students in the prepregnancy period, plan information and education, and conduct further research on the fear of childbirth.

## 1. Introduction

Pregnancy is a multidimensional and unique experience that includes both positive and negative emotions [1]. Fear and anxiety are caused by childbirth, the effects of which are unknown and uncertain [2]. Fear of childbirth may be influenced by sociodemographic, obstetric, and psychosocial factors, such as the woman's age, marital status, education level, income level, parity, gestational week, childbirth pain, previous childbirth experiences, partner support, social support, self-efficacy, post-traumatic stress disorder, low self-esteem, childhood abuse, anxiety, and ability to cope with this situation [3]. Fear of childbirth during pregnancy has been linked to mood disorders, such as anxiety and stress, and negative coping behaviors, such as extreme distress and avoidance [4, 5]. Women with anxiety or depression are twice more likely to be afraid of childbirth, and those with both anxiety and depression are 11 times more likely to be afraid of childbirth [6, 7].

It has been determined that pregnant women who have negative personality traits and have difficulty coping with stress experience fear of childbirth more severely [8]. Women with neurotic personalities, which are characterized by disturbing thoughts that cause emotional distress, have higher levels of fear of childbirth, whereas those with high self-confidence and an extroverted personality type cope better with childbirth pain [9, 10]. Women with neurotic personality types and depressed moods are more afraid of childbirth [11]. It was emphasized that women who were afraid of childbirth were more worried, irritable, and had fewer social interactions than others [12].

Fear of childbirth affects not only pregnant women but also young individuals planning a future pregnancy [13]. It was found that 13.6% of young women before pregnancy were found to have a high level of fear of childbirth [14]. Young women who reported a high level of fear of childbirth were approximately four times more likely to prefer cesarean section [15].

In a study evaluating university students' attitudes towards childbirth, fear of childbirth and perceived high-risk levels were found to affect their preference for cesarean sections [16]. According to a study conducted with university students, students with high levels of fear of childbirth defined birth as frightening and painful and considered obstetric interventions to make birth more manageable, whereas those with low levels of fear regarded birth as a natural event and were more critical of interventions, and students in both groups supported women's autonomous birth care decisions [17]. In another study about university students' attitudes toward vaginal birth and cesarean section, it was discovered that female students had a significant fear of childbirth due to discomfort and worry of harm to their bodies [18].

Understanding the association between fear of childbirth and personality traits, depression, anxiety, and stress in young women who have not given birth can be useful for creating interventions to avoid or minimize the fear of childbirth [15]. However, research on the association between prepregnancy fear of childbirth and personality type, stress, anxiety, and depression levels among university students is scarce. By evaluating young women's stress, anxiety, and depression levels, personality traits, and fear of childbirth levels prior to pregnancy, information and training can be planned, and interviews on birth perceptions and birth preferences can be undertaken.

The aim of this study is to investigate personality traits, stress, anxiety, depression levels, fear of childbirth, and affecting factors in Turkish female university students.

### 1.1. Research Questions

1. What are the personality traits of Turkish university students?
2. What are the levels of stress, anxiety, and depression in Turkish university students?
3. What is the level of fear of childbirth in Turkish university students?
4. What are the factors that affect personality traits, stress, anxiety, depression, and fear of
5. childbirth in Turkish university students?
6. Is there a relationship between personality traits, stress, anxiety, depression levels, and fear of childbirth in Turkish university students?
7. What are the predictors of fear of childbirth in Turkish university students?

## 2. Materials and Methods

The population of this descriptive and correlational study consisted of 465 female students studying at the Faculty of Health Sciences and 1073 female students studying at the Faculty of Education of a university in the Black Sea Region of Turkey.

A total of 500 female students who agreed to participate in the study and met the inclusion criteria were randomly included in the study sample, 264 from the Faculty of Health Sciences and 236 from the Faculty of Education. A sample with a known universe was calculated for the sample, and the prevalence of fear of childbirth in the study of Gökçe İsbir et al. [16] was taken as a reference, as 42.4% (*p* = 0.42).

Sample calculation with a known universe:

  $$\begin{array}{c} n={\text{Nt}}^{2}\text{pq}/d^{2}\left(N-1\right), \end{array}$$

*N*: Number of individuals in the population

*n*: Number of individuals to be sampled

*p*: Frequency of the occurrence of the investigated event (0.42)

*q*: Frequency of the nonoccurrence of the investigated event (0.58)

*t*: Theoretical value found from the *t* table at a certain degree of freedom and detected an error level (1.96)

*d*: The desired deviation according to the incidence of the event (0.05).

It was calculated as *n* = 1538 × (0.42) × (0.58) × (3.84)/1537 × (0.0025) = 374. However, more participants were included in the study due to the possibility of leaving the study. The study was completed with 500 students.

*Inclusion Criteria*

Being 18 years old or older, being female, having no previous pregnancy experience, and agreeing to participate in the study.

*Exclusion Criteria*

Accepting to participate in the study but withdrawing at any stage of the study.

### 2.1. Data Collection

Data were collected via face-to-face interviews between March 1, 2022, and May 28, 2022. A personal information form, the Childbirth Fear–Prior to Pregnancy (CFPP) scale, the Type D Personality Scale (negative affectivity and social inhibition subscales), and the Depression Anxiety Stress Scale-21 (DASS-21) were used to collect the data. The forms and scales were filled in by the students under the supervision of the researchers. Data collection took approximately 15 min.

#### 2.1.1. The Personal Information Form

The form, developed by the researchers after reviewing the literature, consists of questions about women's age, educational status, marital status, their own mode of birth, how they want to give birth, the mode of birth they hear most frequently, family history of a difficult birth, and their information sources about birth [13, 19].

#### 2.1.2. CFPP Scale

The CFPP scale was developed by Stoll et al. [14] to measure the fear of childbirth prior to birth in young women and men. The Likert-type scale, whose validity and reliability study was conducted by Uçar and Taşhan [20], has 10 items. The scoring of the responses is as follows: 1 “strongly disagree”, 2 “disagree”, 3 “partially disagree”, 4 “partially agree”, 5 “agree”, and 6 “strongly agree”. A minimum of 10 and a maximum of 60 points can be obtained from the scale. High scores indicate a high level of fear. Cronbach's alpha value is 0.86 for the original study and 0.91 for this study [20].

#### 2.1.3. The Type D Personality Scale

The Type D Personality Scale was developed by Denollet and adapted into Turkish by Öncü and Vayısoğlu [21, 22]. The 14-item Likert-type scale, each consisting of seven items based on the subjective evaluation of individuals, has “negative affectivity” (2, 4, 5, 7, 9, 12, and 13) and “social inhibition” (1, 3, 6, 8, 10, 11, and 14) subscales and items 1 and 3 are reverse coded. Each statement is answered as “wrong, partly wrong, undecided, partly correct, and correct” and scored between 0 and 4. The subscales can take values between 0 and 28. The cut-off point of the subscales is ≥10. Participants are considered to have type D personality traits if their total score for both subscales is 10 or above [21]. Cronbach's alpha coefficient was found to be *α* = 0.85 for negative affectivity and *α* = 0.76 for social inhibition in the original study and 0.87 and 0.83 for this study, respectively.

#### 2.1.4. The DASS-21

The Turkish validity and reliability study of the DASS-21 [23], which was created by Lovibond and Lovibond by selecting some items in the DASS-42 to shorten the application time, was conducted by Sarıçam [24]. The scale, which includes seven statements regarding depression, anxiety and stress subdimensions, is scored between 0 and 3. The subscales can take values between 0 and 21. The scores obtained from the subscales determine the levels of depression, anxiety, and stress as normal, mild, severe, and very severe. The scoring of the scale is as follows: 0–4 points: normal depression, 5–6 points: mild depression, 7–10 points: moderate depression, 11–13 points: severe depression, and 14 and above points: very severe depression. 0–3 points: normal anxiety, 4–5 points: mild anxiety, 6–7 points: moderate anxiety, 8–9 points: severe anxiety, and 10 and above points: very severe anxiety. A score of 0–7 is defined as normal stress, 8–9 as mild stress, 10–12 as moderate stress, 13–16 as severe stress, and 17 and above as very severe stress. Cronbach's alpha reliability coefficient was found to be *α* = 0.87 for the depression scale, *α* = 0.85 for the anxiety subscale, and *α* = 0.81 for the stress subscale in the original study, and 0.85 for the depression subscale, 0.81 for the anxiety subscale, and 0.80 for the stress subscale in this study.

### 2.2. Data Evaluation

Descriptive statistical methods, including frequency, percentages, mean, standard deviation, and minimum and maximum values, were used to evaluate the descriptive data of the study. The Kolmogorov–Smirnov test, histogram graph, normal distribution curve, skewness, and kurtosis coefficients were used to determine whether the research data fit the normal distribution. Since the research data did not conform to the normal distribution, the differences between the groups were evaluated with nonparametric tests, such as the Mann–Whitney *U* test and the Kruskal–Wallis test. Tamhane and least significant difference (LSD) tests were used to determine from which group the statistical differences originated. The relationships between the scale scores were evaluated with the Spearman correlation analysis test. Linear regression analysis was also used. The level of statistical significance was considered *p*  < 0.05.

### 2.3. Ethical Considerations

Before starting the study, permission to use the scales was sought by email from the authors. The students who took part in the study provided written and verbal consent. Approval was obtained from Ordu University Noninvasive Clinical Research Ethics Committee (17.12.2021-273), and written permission was obtained from the Ordu University Faculty of Health Sciences and Faculty of Education. The study was carried out in accordance with the principles of the Helsinki Declaration.

## 3. Results

According to the results, 52.8% of the students involved in the study were from the Faculty of Health Sciences, 48.4% were nursing students, 30.6% were first-year students, 1% were married, 9.2% had a chronic disease, and 3.8% had a mental illness (Table 1). 73.6% were born with a normal birth, 66.8% were positively told about their own birth, and 75.6% wanted to have a normal birth. 55.8% had most frequently heard of cesarean sections; 37.2% had a family history of a difficult birth; 26% had a family history of a difficult cesarean section, and 28.6% had a family history of a difficult normal birth (Table 2).

The mean scores of the scales used in the study were as follows: CFPP 40.14 ± 11.35, negative affect 12.01 ± 6.79, social inhibition 9.58 ± 6.09, DASS-21 depression 5.58 ± 4.50, DASS-21 anxiety 5.28 ± 4.09, and DASS-21 stress 6.75 ± 4.05 (Table 1).

A moderate positive correlation was found between the negative affectivity and social inhibition subscales of the Type D Personality Scale (*r* = 0.490). Additionally, negative affectivity showed a weak positive correlation with the DASS-21 stress subscale (*r* = 0.225) and anxiety subscale (*r* = 0.273), while it had a moderate positive correlation with the depression subscale (*r* = 0.333) (*p*s  < 0.001, Table 3). Furthermore, a strong positive correlation was observed between the negative affectivity subscale of the Type D Personality Scale and the DASS-21 stress (*r* = 0.574), anxiety (*r* = 0.504), and depression (*r* = 0.619) subscales (*p*s  < 0.001, Table 3).

Table 4 showed that there was only a relationship between students' fear of childbirth prior to pregnancy and negative affectivity (*R* = 0.284, *R*^2^ = 0.080, *F* = 8.648, *p*  < 0.001). These variables explained 10% of the variance in fear of childbirth prior to pregnancy. According to the standardized regression coefficient (*β*), the relative order of significance of the predictor variables on fear of childbirth prior to pregnancy is negative affectivity (*β =* 0.156), anxiety (*β* = 0.085), stress (*β =* 0.078), depression (*β =* 0.039), and social inhibition (*β =* 0.001). The *t*-test results regarding the significance of the regression coefficients were analyzed, and negative affectivity (*p*  < 0.001) was found to be a significant predictor of fear of childbirth before pregnancy. Social inhibition, stress, anxiety, and depression had no significant effect on fear of childbirth prior to pregnancy (*p*s  > 0.05).

**Figure 1:**
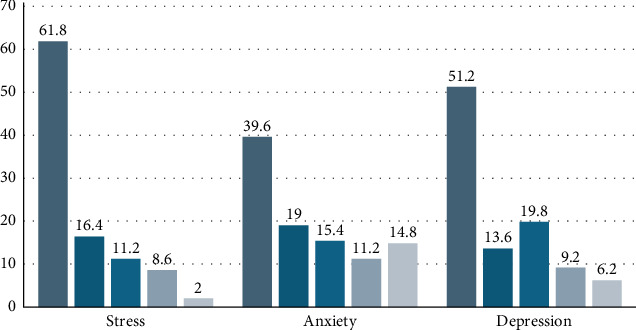
Rates of Depression Anxiety Stress Scale by cut-off scores (%).

**Figure 2:**
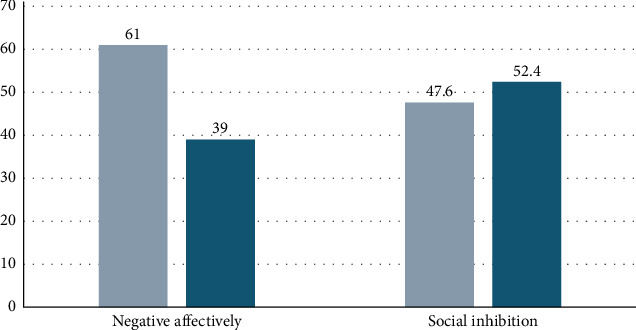
Rates of Type D Personality Scale by cut-off scores (%).

According to the study results, 8.6% of the students exhibited high levels of stress, 14.8% experienced severe anxiety, and 19.8% showed moderate depressive symptoms (Figure 1). Additionally, 61% of the students were found to have negative affectivity, while 47.6% exhibited social inhibition as a personality trait (Figure 2).

No statistically significant difference was found between the socio-demographic characteristics of the students based on their mean scores for CFPP (*p*  > 0.05, Table 1). However, students whose mothers described their birth experiences negatively, those who preferred cesarean delivery, those with a family history of difficult childbirth, and those aware of difficult birth experiences in their social environment had higher CFPP mean scores compared to other students. The differences between these groups were found to be statistically significant (*p*  < 0.05, Table 2).

No statistically significant differences were found between socio-demographic characteristics based on the mean scores of the negative affectivity and social inhibition subscales of the Type D Personality Scale (*p*  > 0.05, Table 1). However, the differences in negative affectivity and social inhibition mean scores were found to be statistically significant among students whose birth stories had never been shared with them and those who preferred cesarean delivery in the future (*p*  < 0.05, Table 2).

The mean scores of the depression subscale of the DASS-21 were found to be higher among students studying Turkish language teaching, first-year students, unmarried students, and those with a mental illness compared to other students, with statistically significant differences between the groups (*p*  < 0.05, Table 1). The mean scores of the anxiety subscale of the DASS-21 were significantly higher among first-year students, those with a chronic illness, and those with a mental illness compared to other students (*p*  < 0.05, Table 1). The mean scores of the stress subscale of the DASS-21 were significantly higher among students with a mental illness compared to others (*p*  < 0.05). However, no significant differences were found in stress scores based on other socio-demographic characteristics (*p*  > 0.05, Table 1). Students who were born via cesarean section and those who had been exposed to more cesarean birth stories in their social environment had significantly higher stress subscale scores. Additionally, students whose own birth stories had not been shared with them and those who preferred cesarean birth had significantly higher stress and depression scores. Furthermore, students with a family history of difficult childbirth and those who had heard of difficult cesarean birth experiences in their environment had significantly higher stress, anxiety, and depression scores (*p*  < 0.05, Table 2). Students who had been exposed to stories of difficult vaginal births in their environment had significantly higher anxiety and depression scores compared to other students (*p*  < 0.05, Table 2). However, no significant differences were found in students' stress, anxiety, and depression scores based on other pregnancy and birth-related characteristics (*p*  > 0.05, Table 2).

## 4. Discussion

In this study, which was performed to investigate the personality traits, stress, anxiety, depression levels, fear of childbirth, and affecting factors in Turkish female university students, the fear of childbirth of the students was found to be above the moderate level (40.14 ± 11.35). In Güleç Şatır's study [13], fear of childbirth in nursing students was shown to be above the middle level. In another study, similar to this one, fear of childbirth before pregnancy was reported to be above the moderate level among healthcare professionals who were planning to have children in the future [25]. Students who have not yet had a pregnancy may feel weak and helpless owing to fears of being harmed during childbirth or experiencing pain that they cannot bear, and this situation may produce a fear of childbirth associated with a prospective future birth experience.

A significantly positive relationship was found between fear of childbirth prior to pregnancy and the subscales of negative affectivity, stress, anxiety, and depression in this study (*p*s  < 0.01). It has been noted that individuals with type D personality traits exhibit high levels of health-related anxiety and are more sensitive to stressful life events [26]. Therefore, it is likely that individuals with this personality trait experience more intense fear of childbirth. It has been suggested that those who experience fear of childbirth prior to pregnancy tend to have greater anxiety about pregnancy and develop negative expectations about childbirth, which negatively impacts their psychological well-being [18]. Anxiety, one of the most common determinants of fear of childbirth, as well as stress and depression, which increase fear of childbirth, are noted to affect fear of childbirth not only during pregnancy but also in the prepregnancy period [18, 27, 28]. Fear of childbirth is likely influenced by characteristics that affect the individual's attitude towards life and negative emotional states.

Positive relationships have been found between the subscales of type D personality, namely negative affectivity and social inhibition, and the subscales of the DASS-21, including stress, anxiety, and depression (Table 1). It is known that individuals with type D personality tend to have poor emotional regulation skills and struggle more with coping with stress [26]. Type D personality is considered a significant risk factor for depression, with individuals exhibiting a tendency to develop persistent negative emotions [29]. Similarly, it has been identified that these individuals are prone to feeling consistently anxious and worried, often responding excessively to situations involving uncertainty and stress [30]. Therefore, it can be concluded that individuals with type D personality are at risk for depression, anxiety, and stress.

In this study, negative affectivity was a predictor of students' fear of childbirth prior to pregnancy (*p*  < 0.05). Dursun et al. discovered that people with neurotic personalities had a high level of fear of childbirth, while Calpbinici et al. discovered a positive association between neurotic personality traits and fear of childbirth [31, 32]. According to Gönenç et al. [33], pregnant women's anxiety about childbirth increased as their neuroticism grew. Literature has citations that personality type influences fear of childbirth [33–35]. Personality traits are believed to affect attitudes throughout pregnancy and birth, and some dominant personality traits may be harmful [36]. Mood changes experienced during pregnancy can cause increased pain and anxiety during the birth process [37]. As is known, personality type affects the coping mechanism in stressful or negative situations [38]. As seen in the study, it is an expected result that individuals with negative emotions in an unpredictable and stressful process, such as birth, have high prepregnancy birth fears.

In this study, students' prepregnancy fear of childbirth did not differ according to their faculty, department, class, marital status, chronic illness, mental illness, or mode of birth. Although the students' socio-demographic characteristics varied, it is believed that they did not influence their fear of childbirth, as they had not yet experienced pregnancy. Similarly, in the study conducted by Dursun et al. [31], no significant difference was found in the fear of childbirth scores of the pregnant women participating in the study based on their age, education level, or family type. Another study indicated that young women with a secondary school education level had higher levels of fear of childbirth [39]. The results of the study demonstrate that fear of childbirth can occur regardless of socio-demographic characteristics [25, 31].

The mean prepregnancy fear of childbirth scores of students who preferred cesarean section, had a family history of difficult births, had someone in their close environment with a history of problematic cesarean sections, and whose own birth story was told negatively were found to be high, with statistically significant differences between the groups. Hauck et al. [40] found that students who had negative experiences and stories about pregnancy and childbirth from family members, as well as those who wished to give birth by cesarean section in the future, exhibited high levels of fear of childbirth. Serçekuş et al. [41] reported that women's negative birth experiences resulted in high levels of fear of childbirth, leading them to prefer cesarean sections. Previous studies also indicate that a high level of fear of childbirth influences preferences for cesarean sections [15, 42, 43]. It is believed that hearing negative birth experiences may trigger anxiety about the possibility of similar experiences occurring, thereby leading to fear of childbirth.

In this study, it was found that the mean scores of the type D personality subscales based on the students' socio-demographic characteristics did not differ, but the “negative affectivity” and “social inhibition” subscale scores of those who had never heard a birth story and those who wished to give birth by cesarean section in the future showed statistically significant differences between the groups (*p*s  < 0.05). Having knowledge of one's own birth story may lead to a sense of deficiency in identity development and may direct the individual towards introspection, thereby influencing personality development [44]. Since personality type and personal characteristics affect psychological resilience [35], fear of childbirth and perception of birth are also influenced. Calpbinici et al. [32] found a negative relationship between fear of childbirth and being extroverted, and a positive relationship between fear of childbirth and neurotic personality traits. Dursun [31] discovered that pregnant women with neurotic personalities had higher levels of fear of childbirth, whereas pregnant women with extroverted personalities experienced less fear. Personality factors were found to influence fear of childbirth and birth preferences, and the cesarean section choices of students with negative affectivity and social inhibition supported the literature [34, 45, 46].

It was determined that the mean scores of DASS-21 depression, anxiety, and stress subscales were influenced by some of the students' socio-demographic characteristics (*p*s  < 0.05, Table 1). Since the students' changing socio-demographic characteristics affect their lifestyle and quality of life, changes in their negative emotional states are to be expected. Similarly, in this study, depression and anxiety scores were found to be high in students who did not engage in physical activity and had poor academic success, while anxiety and stress scores were found to be high in those with chronic illnesses [47]. In the study by Abed et al. [48], it was determined that students' depression, stress, and anxiety scores were affected by socio-demographic characteristics such as gender, class, and economic status.

A significant difference was found between the groups in the DASS-21 subscale scores of students who were born by cesarean section, wished to give birth by cesarean section, heard the most cesarean birth stories, and had a family or close environment history of difficult births (*p*s  < 0.05, Table 2). Malata and Chirwa [49] discovered that negative birth experiences heard from the surroundings increased women's anxiety and fear. Previous bad or traumatic birth experiences are said to be a major cause of fear of childbirth, and fear of childbirth, along with negative emotional states, can influence birth preferences [50–52]. Negative birth experiences, whether from their own birth or from their environment, affect students' negative emotions, make coping strategies more difficult, and generate fear and worry about childbirth.

### 4.1. Strengths, Limitations, and Directions

Although studies on fear of childbirth are widespread, there is a limited number of studies in the literature focusing on the prepregnancy period [20, 53–55]. The results of the present study are very valuable in terms of determining the fear of childbirth in future mothers, the vast majority of whom are single. Additionally, the study provides important findings regarding prepregnancy fear of childbirth, personality type, depression, anxiety, and stress levels.

There are several limitations to this study. The first limitation is that the students included in the study were enrolled at two faculties of a single university. The second limitation is that, due to participation being based on meeting the criteria and voluntary consent, a very large sample size could not be achieved. Therefore, it can serve as an important reference for future studies that will be conducted with a larger sample size and focus on the fear of childbirth and its related factors. The third limitation is that the study was cross-sectional and covered data from the sample group at a specific point in time. The fourth limitation is that the research questions did not ask whether university students had experienced childhood trauma, which could potentially affect their perceptions of childbirth. The fifth limitation is the potential recall bias due to the personal information form and scales being based on self-reports and containing information about the past.

There is a need for screening and intervention studies on this subject. For these reasons, the results of the study cannot be generalized. Future studies are recommended to focus on evidence-based interventions that can address students' fear of childbirth, personality type, depression, anxiety, and stress levels.

## 5. Conclusion

The findings of this study demonstrated that students reported above-moderate levels of fear of childbirth prior to pregnancy and that traits, anxiety, depression, and stress contributed to enhancing the fear of childbirth. These results are believed to be valuable for healthcare planning for future mothers. This study found a significant positive relationship between prepregnancy fear of childbirth and the subscales of negative affectivity, stress, anxiety, and depression. This result serves as evidence that students' personality type and psychological state are determinants of prepregnancy fear of childbirth. Negative affect is a predictor of students' fear of childbirth before pregnancy, and this variable explains their fear of childbirth. Early identification of the factors leading to fear of childbirth and interventions aimed at resolving them can reduce negative pregnancy and childbirth experiences. Therefore, it is recommended to plan interventions that strengthen students' mental states and coping mechanisms according to their personality characteristics and to conduct studies with high levels of evidence on fear of childbirth.

## Figures and Tables

**Table 1:** Students' mean scores according to their socio-demographic characteristics.

Characteristics	*n*	%	Childbirth fear–prior to pregnancy mean ± SD	Negative affectivity mean ± SD	Social inhibition mean ± SD	DASS-21 stress mean ± SD	DASS-21 anxiety mean ± SD	DASS-21 depression mean ± SD
Faculty								
Faculty of health sciences	264	52.8	40.20 ± 10.89	12.07 ± 6.99	9.66 ± 6.02	6.57 ± 3.88	5.16 ± 3.90	5.70 ± 4.27
Faculty of education	236	47.2	40.07 ± 11.87	11.95 ± 6.57	9.49 ± 6.19	6.94 ± 4.23	5.41 ± 4.31	5.45 ± 4.74
Test and *p*	—	—	*U* = 35,757 *p* = 0.806	*U* = 31140, *p* = 0.994	*U* = 30350, *p* = 0.619	*U* = 29827, *p* = 0.410	*U* = 30713, *p* = 0.785	*U* = 28988, *p* = 0.178
Department								
Nursing^a^	242	48.4	40.02 ± 10.89	12.18 ± 7.03	9.77 ± 5.97	6.48 ± 3.82	5.09 ± 3.90	5.76 ± 4.33
Health management ^b^	22	4.4	42.18 ± 10.86	10.81 ± 6.63	8.40 ± 6.56	7.63 ± 4.49	5.86 ± 3.97	5.04 ± 3.56
Elementary mathematics teaching^c^	65	13.0	40.07 ± 10.84	12.81 ± 6.21	11.26 ± 6.06	6.83 ± 3.31	5.18 ± 3.70	5.44 ± 4.58
Science teaching^d^	2	0.4	35.50 ± 2.12	13.50 ± 4.94	12.50 ± 12.02	5.50 ± 2.12	3.50 ± 0.70	3.00 ± 0.00
Classroom teaching^e^	56	11.2	39.07 ± 12.90	10.62 ± 7.00	8.07 ± 6.14	7.35 ± 5.31	5.44 ± 4.83	4.94 ± 4.79
Preschool teaching^f^	38	7.6	37.57 ± 12.82	11.18 ± 7.45	9.60 ± 5.93	5.86 ± 4.01	4.81 ± 4.03	4.21 ± 4.55
Turkish teaching^g^	16	3.2	37.50 ± 13.19	12.50 ± 4.99	7.50 ± 5.24	5.00 ± 2.80	3.62 ± 3.11	5.56 ± 3.81
Social sciences teaching^h^	17	3.4	43.64 ± 11.30	16.23 ± 6.75	9.94 ± 7.22	8.41 ± 4.63	7.11 ± 4.74	8.94 ± 5.72
PCG^j^	42	8.4	43.47 ± 10.53	11.07 ± 5.60	8.97 ± 6.00	7.78 ± 4.14	6.38 ± 4.75	5.95 ± 4.62
Test and *p*	—	—	KW = 10.27, *p* = 0.246	KW = 11.67, *p* = 0.166	KW = 12.08, *p* = 0.148	KW = 12.36, *p* = 0.136	KW = 9.58 *p* = 0.295	**KW = 16.26**, **p** = 0.039**between** ***a–d/c–d/*** ***d–h/d–j***
Class								
1. Year^a^	153	30.6	40.60 ± 10.21	12.83 ± 7.15	10.58 ± 6.57	6.97 ± 4.15	6.02 ± 4.21	6.46 ± 4.83
2. Year^b^	121	24.2	39.76 ± 12.74	12.05 ± 6.46	9.46 ± 5.56	6.28 ± 3.47	4.73 ± 3.64	5.31 ± 3.92
3. Year^c^	145	29.0	40.46 ± 11.34	11.53 ± 6.72	9.22 ± 6.14	6.86 ± 4.40	5.23 ± 4.15	5.33 ± 4.56
4. Year^d^	81	16.2	39.28 ± 12.15	11.25 ± 6.67	8.50 ± 5.66	6.83 ± 4.03	4.79 ± 4.29	4.79 ± 4.38
Test and *p*	—	—	KW = 0.656, *p* = 0.884	KW = 3.20, *p* = 0.361	KW = 5.91, *p* = 0.11	KW = 0.980, *p* = 0.806	**KW = 9.56**, **p** = 0.023 **between** ***a–b***	**KW = 9.27**, **p** = 0.026 **between** **a–d**
Marital status								
Married	5	1.0	45.80 ± 8.40	6.60 ± 6.84	7.00 ± 6.00	3.80 ± 4.08	3.40 ± 3.36	2.40 ± 4.82
Single	495	99.0	40.08 ± 11.37	12.07 ± 6.78	9.60 ± 6.10	6.78 ± 4.04	5.30 ± 4.10	5.62 ± 4.49
Test and *p*	—	—	*U* = 923, *p* = 0.328	*U* = 696, *p* = 0.09	*U* = 925, *p* = 0.331	*U* = 706, *p* = 0.09	*U* = 887, *p* = 0.274	***U* = 549**, **p** = 0.032
Chronic disease								
Yes	46	9.2	38.80 ± 12.51	12.21 ± 6.55	8.93 ± 6.30	7.76 ± 4.08	6.89 ± 3.97	6.54 ± 4.74
No	454	90.8	40.27 ± 11.23	11.99 ± 6.82	9.64 ± 6.08	6.65 ± 4.04	5.12 ± 4.08	5.49 ± 4.47
Test and *p*	—	—	*U* = 100, *p* = 0.686	*U* = 101, *p* = 0.770	*U* = 968, *p* = 0.414	*U* = 870, *p* = 0.063	***U* = 753**, **p** = 0.00	*U* = 904, *p* = 0.133
Mental illness								
Yes	19	3.8	41.00 ± 13.56	14.78 ± 6.46	9.94 ± 7.12	9.21 ± 5.20	9.15 ± 5.38	8.31 ± 5.17
No	481	96.2	40.11 ± 11.27	11.90 ± 6.79	9.56 ± 6.06	6.65 ± 3.97	5.13 ± 3.97	5.48 ± 4.44
Test and *p*	—	—	*U* = 430, *p* = 0.673	*U* = 340, *p* = 0.059	*U* = 450, *p* = 0.912	***U* = 323**, **p** = 0.03	***U* = 246**, **p** = 0.00	***U* = 296**, **p** = 0.00
Total	**500**	**100.0**	**40.14 ± 11.35**	**12.01 ± 6.79**	**9.58 ± 6.09**	**6.75 ± 4.05**	**5.28 ± 4.09**	**5.58 ± 4.50**

*Note*: Alphabetical superscripts (a,b,c,d,e,f,g,h,j) are used to identify groups. Statistically significant values are shown in bold.

**Table 2:** Students' mean scores according to their pregnancy and birth information.

Characteristics	*n*	%	Childbirth fear–prior to pregnancy mean ± SD	Negative affectivity mean ± SD	Social inhibition mean ± SD	DASS-21 stress mean ± SD	DASS-21 anxiety mean ± SD	DASS-21 depression mean ± SD
What was your own mode of birth?								
Normal	368	73.6	40.25 ± 11.29	11.74 ± 6.97	9.57 ± 6.19	6.48 ± 3.95	5.15 ± 4.04	5.44 ± 4.35
Cesarean	112	26.4	39.84 ± 11.56	12.77 ± 6.24	9.60 ± 5.85	7.51 ± 4.23	5.64 ± 4.24	5.99 ± 4.89
Test and *p*	—	—	*U* = 236, *p* = 0.656	*U* = 221, *p* = 0.131	*U* = 239, *p* = 0.820	***U* = 209**, **p** = 0.01	*U* = 225, *p* = 0.215	*U* = 230, *p* = 0.379
How did your mother tell you your birth story?								
Positive^a^	334	66.8	39.20 ± 11.42	11.27 ± 6.84	9.10 ± 6.03	6.43 ± 4.04	5.00 ± 4.04	5.21 ± 4.38
Negative^b^	108	21.6	42.31 ± 11.26	13.30 ± 6.38	9.89 ± 5.35	7.25 ± 3.71	5.75 ± 3.92	6.21 ± 4.44
No birth history^c^	58	11.6	41.51 ± 10.54	13.87 ± 6.66	11.74 ± 7.26	7.68 ± 4.50	6.00 ± 4.58	5.07 ± 5.07
Test and *p*	—	—	**KW = 8.41**, **p** = 0.01 **between *a–b***	**KW = 12.94**, **p** = 0.00 **between** ***a*–*b* (*a–c*)**	**KW = 7.99**, **p** = 0.01 **between** ***a*–*c***	**KW = 7,59**, **p** = 0.02 **between** ***a*–*c***	KW = 5.94, *p* = 0.05	**KW = 7.51**, **p** = 0.02 **between** ***a*–*b (a–c)***
How would you like to give birth?								
Normal	378	75.6	39.11 ± 11.35	11.42 ± 6.57	9.05 ± 5.87	6.47 ± 3.94	5.10 ± 3.94	5.24 ± 4.31
Cesarean	122	24.4	43.33 ± 10.70	13.83 ± 7.17	11.22 ± 6.48	7.60 ± 4.29	5.85 ± 4.50	6.66 ± 4.91
Test and *p*	—	—	*U* = 180, **p** = 0.00	*U* = 185, **p** = 0.00	*U* = 187, **p** = 0.00	*U* = 195, **p** = 0.01	*U* = 210, *p* = 0.15	***U* = 192**, **p** = 0.00
What type of birth do you hear most often?								
Normal	221	44.2	40.06 ± 11.69	11.59 ± 6.94	9.47 ± 6.13	6.27 ± 3.98	5.24 ± 4.26	5.47 ± 4.57
Cesarean	279	55.8	40.20 ± 11.09	12.34 ± 6.66	9.66 ± 6.08	7.13 ± 4.07	5.31 ± 3.97	5.67 ± 4.45
Test and *p*	—	—	*U* = 307, *p* = 0.96	*U* = 286, *p* = 0.17	*U* = 301, *p* = 0.68	***U* = 268**, **p** = 0.01	*U* = 299, *p* = 0.57	*U* = 296, *p* = 0.44
Do you have a family history of difficult birth?								
Yes	186	37.2	41.50 ± 10.84	12.67 ± 6.84	9.68 ± 6.22	7.16 ± 3.73	6.02 ± 3.77	6.38 ± 4.34
No	314	62.8	39.33 ± 11.58	11.62 ± 6.74	9.52 ± 6.03	6.51 ± 4.21	4.84 ± 4.22	5.11 ± 4.53
Test and *p*	—	—	***U* = 257**, **p** = 0.02	*U* = 267, *p* = 0.11	*U* = 289, *p* = 0.88	***U* = 253**, **p** = 0.01	***U* = 227**, **p** = 0.00	***U* = 232**, **p** = 0.00
Is there anyone in your circle with a difficult cesarean birth history?								
Var	130	26.0	41.85 ± 11.32	12.35 ± 6.36	9.58 ± 6.26	7.34 ± 3.80	6.33 ± 4.25	6.18 ± 4.34
Yok	370	74.0	39.54 ± 11.31	11.89 ± 6.94	9.58 ± 6.04	6.54 ± 4.12	4.91 ± 3.98	5.37 ± 4.54
Test and *p*	—	—	***U* = 209**, **p** = 0.02	*U* = 230, *p* = 0.48	*U* = 239, *p* = 0.92	***U* = 209**, **p** = 0.02	***U* = 191**, **p** = 0.00	***U* = 207**, **p** = 0.02
Is there anyone in your circle with a difficult normal birth history?								
Var	143	28.6	41.84 ± 10.22	12.44 ± 6.71	10.27 ± 6.80	7.19 ± 4.16	6.27 ± 4.12	6.39 ± 4.59
Yok	357	71.4	39.46 ± 11.71	11.84 ± 6.83	9.30 ± 5.77	6.57 ± 4.00	4.88 ± 4.02	5.26 ± 4.43
Test and *p*	—	—	***U* = 226**, **p** = 0.04	*U* = 241, *p* = 0.33	*U* = 237, *p* = 0.21	*U* = 234, *p* = 0.14	***U* = 199**, **p** = 0.00	***U* = 215**, **p** = 0.00

*Note*: Alphabetical superscripts (a,b,c) are used to identify groups. Statistically significant values are shown in bold.

**Table 3:** Correlations between students' fear of birth scale prior to pregnancy, Type D Personality Scale, and Depression Anxiety Stress Scale mean scores.

Scales and subscales	Fear of birth scale prior to pregnancy	Social inhibition	Negative affectivity	DASS-21 stress	DASS-21 anxiety	DASS-21 depression
Fear of birth scale prior to pregnancy	—	—	—	—	—	—
Social inhibition	0.113^a^	—	—	—	—	—
Negative affectivity	0.282^b^	0.490^b^	—	—	—	—
DASS-21 stress	0.241^b^	0.225^b^	0.574^b^	—	—	—
DASS-21 anxiety	0.231^b^	0.273^b^	0.504^b^	0.681^b^	—	—
DASS-21 depression	0.221^b^	0.333^b^	0.619^b^	0.665^b^	0.610^b^	—

^a^Correlation is significant at the 0.05 level.

^b^Correlation is significant at the 0.01 level.

**Table 4:** Predictors of fear of birth prior to pregnancy.

Subscales	*B*	SE	*β*	*t*	*p*-Value
Constant	34.208	1.081	—	31.632	**0.000**
Negative affectivity	3.621	1.194	0.156	3.034	**0.003**
Social inhibition	0.031	1.072	0.001	0.029	0.977
Stress level	0.815	0.650	0.078	1.254	0.210
Anxiety level	0.660	0.458	0.085	1.441	0.150
Depression level	0.343	0.539	0.039	0.637	0.525

*Note*: *R* = 0.284, *R*^2^ = 0.080, *F* = 8.648, *p* = 0.000. Statistically significant values are shown in bold.

## Data Availability

The data that support the findings of this study are available on request from the corresponding author. The data are not publicly available due to privacy or ethical restrictions.
